# Education for primary health care in Africa

**DOI:** 10.4102/phcfm.v15i1.4034

**Published:** 2023-04-06

**Authors:** Robert Mash, Dorothee Van Breevoort, Martha Makwero, Mpundu Makasa, Akye Essuman, Innocent Besigye

**Affiliations:** 1 Division of Family Medicine and Primary Care, Faculty of Medicine and Health Sciences, Stellenbosch University, Cape Town, South Africa; 2 Department of Family Medicine, Kamuzu University of Health Sciences, Blantyre, Malawi; 3 Department of Public Health, Community and Family Medicine Unit, School of Medicine, University of Zambia, Lusaka, Zambia; 4 Department of Family Medicine, University of Health and Allied Sciences, Ho, Ghana; 5 Department of Family Medicine, School of Medicine, Makerere University, Kampala, Uganda

## Introduction

The Primary Care and Family Medicine (PRIMAFAMED) network held a conference in Malawi with the theme of ‘Education for Primary Health Care in Africa’ 18–19 October 2022.^1^ The PRIMAFAMED network has evolved over 20 years to connect departments of family medicine and primary care in sub-Saharan Africa.^2^ The focus is South–South collaboration on education and research, with support from the Global North. The PRIMAFAMED network is also the official academic wing of the World Organization of Family Doctors (WONCA) in Africa. At this conference, we were able to bring together participants from 18 countries and 29 institutions (Table 1).

**TABLE 1:** Participating countries and institutions.

Country	Institutions
Belgium	University of Ghent
Botswana	University of Botswana
Democratic Republic of Congo	Protestant University of Congo
Ethiopia	University of Addis Ababa
Ghana	Ghana College of Physicians & Surgeons
Ireland	College of General Practitioners
Kenya	Aga Khan University
	Kenyatta University
	Moi University
Malawi	Kamuzu University of Health Sciences
	Malawi College of Health Sciences
Namibia	University of Namibia
Nigeria	FMC Keffi, Nasarawa State
Norway	University of Bergen
Somaliland	Amoud University
South Africa	University of Cape Town
	Stellenbosch University
	Free State University
	Walter Sisulu University
	University of KwaZulu-Natal
	Pretoria University
	University of Witwatersrand
Sudan	University of Gezira
Tanzania	Muhimbili University
	Aga Khan University
Uganda	Makerere University
	Mbarara University
Zambia	University of Zambia
Zimbabwe	University of Zimbabwe

FMC, federal medical centre.

## State of postgraduate family medicine education in sub-Saharan Africa

The PRIMAFAMED network has previously rated the development of postgraduate family medicine education in sub-Saharan Africa according to the stages of change model.^3^ In this model, countries (or institutions) can be at different stages of change, with different educational and research needs. The stages are defined as:

- Pre-contemplation: Not thinking about family medicine training in the near future.
- Contemplation: Key stakeholders (educational institutions, department of health, registration body) are debating whether to introduce family medicine training.
- Preparation and action: Family medicine training has started but with no or very few graduates as yet.
- Maintenance: Family medicine training is established and the focus is on further developing and consolidating the programmes.
- Relapse: Family medicine training programmes have collapsed or gone backwards.
- Permanent change: So well established that it is unlikely to relapse.

**FIGURE 1:**
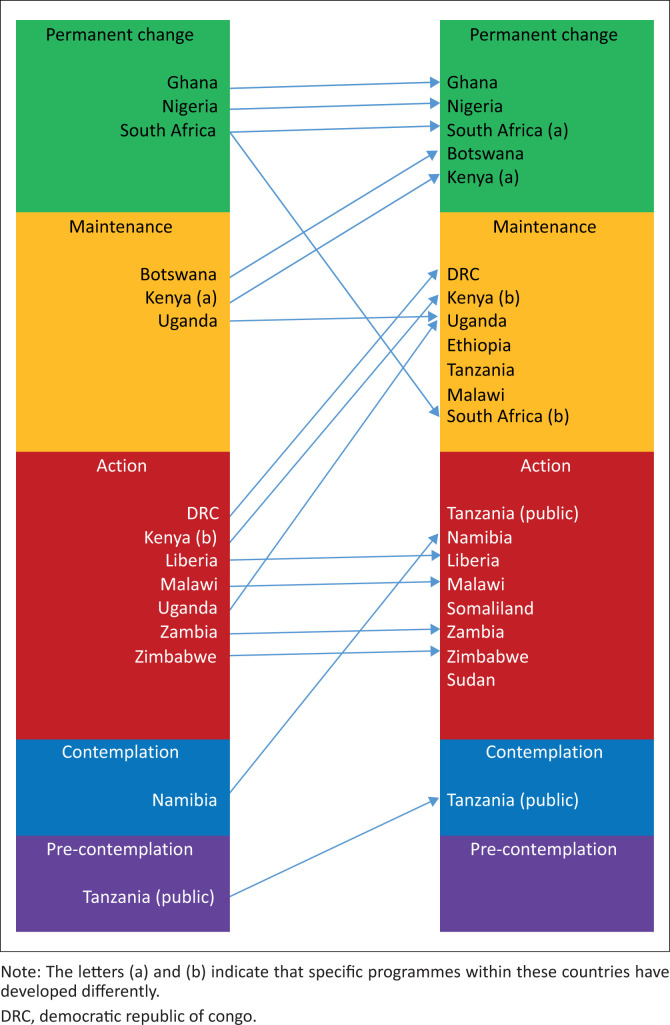
Development of family medicine training by country from 2019 to 2022.

Figure 1 shows how participants self-assessed the development of family medicine training in their countries, and compares their assessment in 2022 with the assessment made in 2019.^3^ In some countries, institutions are at quite different stages. In Tanzania, the private sector training programme is now well established (maintenance), while the public sector programme is planning to start training (contemplation). In Kenya, the five programmes are at different stages with at least two now well established (permanent change) and the others still growing (maintenance).

Namibia had increased its readiness to change and moved from contemplation to action phase. The Democratic Republic of Congo, Kenya and Uganda shifted from action phase to maintenance. The majority of the participants from Malawi and Zambia categorized these countries in the action phase, although others categorized them in the maintenance phase. This might suggest that there is progress in incorporating family medicine into the national health system in these countries. Some participants thought that some training programmes in Somaliland and South Africa might be at risk of relapse. This might indicate that incorporation of family medicine into the health system is dynamic and needs constant advocacy with different stakeholders and alignment to the country’s health needs. Finally, Botswana has shifted from the maintenance stage into permanent change.

From this evaluation together with different presentations and workshops during this PRIMAFAMED meeting, we can acknowledge that several countries showed progress in the development of family medicine and the incorporation into their national health systems. Continuous evaluation, curricula development, faculty development and advocacy are needed to increase the readiness to change and incorporate family medicine into health systems.

## Creating an East Central and Southern Africa College of Family Physicians

Participants supported the idea of creating an East, Central and Southern African College of Family Physicians.^4^ Many other specialist disciplines have already established such colleges to collaborate across countries and ensure an increased supply of new specialists. In PRIMAFAMED, many countries have newly established small-scale postgraduate training programmes, with very few clinical trainers and academic family physicians. It is very hard for these programmes to develop valid and reliable forms of high-stakes licensing examinations, while working separately in siloes. By pooling resources and expertise in a regional college it will be possible to create a regional examination at an international standard with less work for individual departments. Such a college may also be able to help develop training programmes and clinical trainers. The PRIMAFAMED network will set up a working group to explore the idea further and take it forward under the initial leadership of Prof. Sunanda Ray and Prof. Farai Madzimbamuto from Botswana.

## Creating World Health Organization collaborating centres for primary health care

Currently, the World Health Organization (WHO) has 14 collaborating centres for primary health care (PHC) (four in Europe, five in Asia, four in North America). There are none in Africa, and the University of Ghent’s Unit for Family Medicine (itself a collaborating centre) has obtained a grant with Stellenbosch University to develop suitable candidates in the African region.

Dr. Anna Galle attended the conference and presented the opportunity. Such collaborating centres facilitate the set-up of capacity building programmes to strengthen the local health workforce, improve service delivery and disseminate expertise. They also play a role at the global level and can influence the global agenda of health priorities and challenges.

This opportunity is unfortunately limited to certain countries by the Flemish Interuniversity Council who are funding the initiative. These countries are Benin, Burundi, Democratic Republic of Congo, Ethiopia, Kenya, Rwanda, South Africa, Tanzania and Uganda.

## Engaging with planetary health education and research

The PRIMAFAMED network is a founding member of the Climate Change, Migration and Health network.^5^ Dr Charlotte Scheerens (Ghent University) and Dr Christian Lokotola (Stellenbosch University) hosted a workshop to look at how planetary health can be incorporated into education of the PHC and family medicine workforce.

The CliMigHealth network explores the nexus between climate change, migration and health with a focus on research, education and public awareness. Stellenbosch University has also obtained a grant with the University of Ghent to work on climate change and PHC within this broader nexus. An initial scoping review has pointed towards 10 research priorities and there will be opportunities for PRIMAFAMED to collaborate on addressing some of these. In 2022, the *African Journal of Primary Health Care and Family Medicine* published a special collection of short reports on climate change and PHC, which should lead to a number of more in-depth case studies.^6^ The South African Association of Health Educationalists has also set up a special interest group to spearhead educational initiatives.^7^ WONCA is offering a free online course on planetary health for family doctors.^8^

## The Primary Health Care Research Consortium

The PRIMAFAMED network is also a founding member of the Primary Health Care Research Consortium (PHCRC), which is championing low- and middle-income country (LMIC) led research to address global research priorities in PHC systems and services.^9^ The consortium has completed an initial body of work, which will soon be published:

- How are different LMICs implementing PHC team integration to support comprehensive PHC? A multi-country mixed methods study – Mexico, Uganda and India.
- Characterising the Use of Primary Health Care (PHC) Performance Information: A multiple case study in El Salvador, Lebanon and Malawi.
- An International Survey on the Integration of Public Health and Primary Care in coronavirus disease 2019 (COVID-19) Vaccination Campaigns (with Besrour Centre).
- Identifying effective primary health care service delivery models for integrated management of non-communicable chronic diseases in resource-constrained settings (with the Global Alliance on Chronic Diseases).

The Bill and Melinda Gates Foundation was the main funder and unfortunately its new strategy on PHC does not include support for the PHCRC. The consortium is now looking for new funding to take the work forward.

## AfriWon Research Collaboration

The PRIMAFAMED network supported the second cycle of online training and e-mentorship for novice and early career researchers in the field of family medicine in the African region. This initiative has been led by the young family physicians in WONCA (called AfriWon) and had considerable support also from Boston University. The AfriWon Research Collaboration (ARC) developed 10 online modules that help mentees write a research proposal over a period of 6 months. During this period, they are also mentored by a small group that includes a faculty mentor (an experienced researcher) and a peer mentor (a less experienced researcher). The faculty mentor should mentor both the mentee and the peer mentor, as the peer mentor should also develop their capability in postgraduate supervision.

In the second cycle, only 7 out of the 20 mentees completed research proposals, while another seven developed incomplete proposals. Participants came from East, West and Southern Africa. Evaluation suggested the need to improve the management of the mentors, administration and logistics as well as to secure further funding. This remains a potentially useful initiative, especially for training programmes with a lack of postgraduate supervisors and expertise.

## Strengthening education for primary health care in Malawi and Zambia

This conference was largely funded by the Norwegian programme for capacity development in higher education and research for development (NORHED) as part of their project on strengthening health systems through primary care leaders education (PRICE) in Malawi and Zambia. The project is primarily focused on improving education for the PHC and family medicine workforce with higher-quality graduates, higher-quality research and more inclusive higher education. The PRIMAFAMED network is a partner on the project and enables South–South visibility and collaboration for the work carried out in these two countries.

## Applying the World Organization of Family Doctors postgraduate accreditation standards

Prof. Akye Essuman representing the WONCA Working Party on Education (WWPE) gave a plenary talk on the work of the Working Party and in particular the accreditation standards.^10^ The WWPE aims to support high quality education, training, assessment and continuing professional development in general practice and family medicine for medical students, doctors in training, and established general practitioners and family doctors. The accreditation criteria are available on the WONCA website and the Working Party can be approached to formally accredit programmes and give feedback. Prof. Essuman also ran a workshop with participants who might want to use these criteria to strengthen their own programmes.

## Conclusion

The annual PRIMAFAMED meeting was a successful event and participants particularly enjoyed networking, interacting and building new relationships. Everyone had a chance to present an educational innovation or research findings^11^ and to participate in interactive workshops on key issues. The location on the shore of Lake Malawi and the warm hospitality of the people of Malawi was appreciated.
